# Blockade of AMPA Receptor Regulates Mitochondrial Dynamics by Modulating ERK1/2 and PP1/PP2A-Mediated DRP1-S616 Phosphorylations in the Normal Rat Hippocampus

**DOI:** 10.3389/fncel.2019.00179

**Published:** 2019-05-01

**Authors:** Ji-Eun Kim, Hui-Chul Choi, Hong-Ki Song, Tae-Cheon Kang

**Affiliations:** ^1^Department of Anatomy and Neurobiology, College of Medicine, Hallym University, Chuncheon, South Korea; ^2^Institute of Epilepsy Research, College of Medicine, Hallym University, Chuncheon, South Korea; ^3^Department of Neurology, College of Medicine, Hallym University, Chuncheon, South Korea

**Keywords:** calcineurin, JNK, MFN1/2, MK801, NMDA receptor, OPA1, p38 MAPK, protein phosphatase

## Abstract

*N*-Methyl-D-aspartate receptor (NMDAR) and α-amino-3-hydroxy-5-methyl-4-isoxazolepropionic acid receptor (AMPAR) activations induce fast and transient mitochondrial fragmentation under pathophysiological conditions. However, it is still unknown whether NMDAR or AMPAR activity contributes to mitochondrial dynamics under physiological conditions. In the present study, MK801 (a non-competitive NMDAR antagonist) did not affect mitochondrial length in hippocampal neurons as well as phosphorylation levels of dynamin-related protein 1 (DRP1)-serine (S) 616, extracellular-signal-regulated kinase 1/2 (ERK1/2), c-Jun N-terminal kinase (JNK), p38 mitogen-activated protein kinase (p38 MAPK) and AMPAR. In contrast, perampanel (a non-competitive AMPAR antagonist) elongated mitochondrial length in neurons concomitant with diminishing phosphorylations of DRP1-S616, ERK1/2, and JNK, but not p38 MAPK. Perampanel also reduced protein phosphatase (PP) 1, PP2A and PP2B phosphorylations, indicating activations of these PPs which were unaffected by MK801. U0126 (an ERK1/2 inhibitor) elongated mitochondrial length, accompanied by the reduced DRP1-S616 phosphorylation. SP600125 (a JNK inhibitor) did not influence mitochondrial length and DRP1 phosphorylations. Okadaic acid (a PP1/PP2A inhibitor) reduced mitochondrial length with the up-regulated DRP1-S616 phosphorylation, while CsA (a PP2B inhibitor) increased it with the elevated DRP1-S637 phosphorylation. Co-treatment of okadaic acid or CsA with perampanel attenuated the reductions in DRP1-S616 and -S637 phosphorylation without changing DRP1 expression level, respectively. GYKI 52466 (another non-competitive AMPAR antagonist) showed the similar effects of perampanel on phosphorylations of DRP1, ERK1/2, JNK, PPs, and GluR1 AMPAR subunits. Taken together, our findings suggest that a blockade of AMPAR may regulate the cooperation of ERK1/2- and PP1/PP2A for the modulation of DRP1 phosphorylations, which facilitate mitochondrial fusion.

## Introduction

Mitochondria are dynamic organelles responsible for the generation of ATP via oxidative phosphorylation, which is essential for cell viability. To maintain mitochondrial homeostasis, mitochondria change their shapes by mitochondrial dynamics (fusion and fission; Li et al., 2004; Verstreken et al., 2005). Mitochondrial fission (fragmentation) is necessary for the biogenesis of mitochondria and their quality control by mitophagic elimination (Youle and Van der Bliek, 2012). Mitochondrial fusion (elongation) is also involved in mitochondrial quality control by repartition of lipids, proteins and mitochondrial DNA (Nakada et al., 2001; Ono et al., 2001). Therefore, the aberrant mitochondrial dynamics result in the impaired bioenergetics, excessive generation of reactive oxygen species (ROS), loss of mitochondrial membrane potential, dysfunction of endogenous respiration and the release of pro-apoptotic factors from mitochondria, which trigger cell death (Morris and Hollenbeck, 1993; Ligon and Steward, 2000; Weiss et al., 2000; Frank et al., 2001; Olichon et al., 2003; Chen et al., 2005; Youle and Van der Bliek, 2012).

The molecular machinery of mitochondrial fission and fusion involves various proteins, which share reciprocal relationships. Briefly, dynamin-related protein 1 (DRP1) drives mitochondrial division, while mitofusin 1/2 (MFN1/2) and optic atrophy 1 (OPA1) mediate mitochondrial elongation (Westermann, 2008). Recently, it has been reported that glutamate-mediated excitatory transmission affects mitochondrial dynamics through various signaling pathways regulating DRP1 activity under pathophysiological condition (Cereghetti et al., 2008; Han et al., 2008; Jahani-Asl et al., 2011; Nguyen et al., 2011). However, the effects of glutamate on mitochondrial dynamics are distinct from each glutamate receptor subtypes. *N*-methyl-D-aspartate receptor (NMDAR) and α-amino-3-hydroxy-5-methyl-4-isoxazolepropionic acid receptor (AMPAR) activations induce fast and transient mitochondrial fragmentation (Rintoul et al., 2003; Martorell-Riera et al., 2014; Ruiz et al., 2018). In contrast, kainate receptor activation cannot evoke mitochondrial fission (Rintoul et al., 2003). Interestingly, mitochondrial division inhibitor 1 (Mdivi-1) abrogates mitochondrial fragmentation induced by NMDA, but not AMPA (Ruiz et al., 2018). Since NMDAR is permeable to Ca^2+^ (MacDermott et al., 1986) as well as Na^+^ and K^+^ ions (Ascher and Nowak, 1987), but AMPAR prefers Na^+^ to Ca^2+^ influx (Hollmann et al., 1991), it is likely that these distinct properties of NMDAR and AMPAR would activate the different signaling pathways, which lead to the differential phenomena in glutamate-induced mitochondrial dynamics. Therefore, it is noteworthy to investigate the down-stream effectors of each glutamate receptor subtypes regulating mitochondrial dynamics under physiological condition, which remain yet to be explored.

During the course of this study, we validated the effects of a non-competitive NMDAR antagonist (MK801) and non-competitive AMPAR antagonists (perampanel and GYKI 52466) on mitochondrial length in the rat hippocampal neurons to elucidate the receptor-mediated mechanisms underlying the mitochondrial dynamics *in vivo*. Here, we demonstrate that the blockade of AMPAR led to mitochondrial elongations via regulating ERK1/2- and PP1/PP2A-mediated DRP1-S616 phosphorylations. Therefore, our findings suggest that AMPAR activity may contribute to mitochondrial dynamics under physiological condition.

## Materials and Methods

### Experimental Animals and Chemicals

Male Sprague-Dawley (SD) rats (7 weeks old, Daehan Biolink, South Korea) were used in the present study. Animals were given a commercial diet and water *ad libitum* under controlled conditions (22 ± 2°C, 55 ± 5% and a 12:12 light/dark cycle with lights). Animal protocols were approved by the Institutional Animal Care and Use Committee of Hallym University (Chunchon, South Korea). The number of animals used, and their suffering were minimized in all cases. All reagents were obtained from Sigma-Aldrich (St. Louis, MO, United States), except as noted.

### MK801 and Perampanel Trials

MK801 (0.3 mg/kg, i.p., *n* = 14), perampanel (8 mg/kg, i.p., Eisai Korea, Inc., *n* = 14), GYKI 52466 (10 mg/kg, i.p., *n* = 7) or saline (vehicle, *n* = 21) was daily administered at a certain time of the day (PM 6:00) over a 1 week period. In pilot study, these dosages of MK801, perampanel, or GYKI 52466 treatment did not show behavioral and neurological defects in normal animals. After treatments (18 h after the last treatment), animals were used for western blot study and immunohistochemistry.

### Surgery and Chemical Infusions

Under Isoflurane anesthesia (3% induction, 1.5–2% for surgery and 1.5% maintenance in a 65:35 mixture of N_2_O:O_2_), animals were infused with each chemical into the right lateral ventricle (1 mm posterior; 1.5 mm lateral; -3.5 mm depth to the bregma) using a brain infusion kit 1 and an Alzet 1007D osmotic pump (Alzet, Cupertino, CA, United States). The osmotic pump contained (1) vehicle (*n* = 14), (2) U0126 (an ERK1/2 inhibitor, 25 μM; *n* = 14), (3) SP600125 (a JNK inhibitor, 10 μM; *n* = 14), (4) okadaic acid (a PP1/PP2A inhibitor, 10 μM, Cayman, United States; *n* = 21), (5) cyclosporin A (CsA, a PP2B inhibitor, 250 μM; *n* = 21), (6) U0126 + okadaic acid (*n* = 7), or (7) U0126 + CsA (*n* = 7). In pilot study and our previous studies, each compound treatment did not show behavioral and neurological defects in normal animals (Min et al., 2017; Park and Kang, 2018). Some okadaic acid or CsA-infused animals (*n* = 7, respectively) were also given perampanel by the same protocol aforementioned. Seven days after infusion, animals were used for western blot and immunohistochemistry (*n* = 7 in each group, respectively).

### Western Blot

After animals (*n* = 7 in each group, respectively) were sacrificed via decapitation, the hippocampi were obtained. The hippocampal tissues were homogenized and protein concentration determined using a Micro BCA Protein Assay Kit (Pierce Chemical, Rockford, IL, United States). Western blot was performed by the standard protocol. The primary antibodies used in the present study were listed in Table 1. The bands were detected and quantified on an ImageQuant LAS4000 system (GE Healthcare, United States). Since OPA1, ERK1/2 and pERK1/2 antibodies, but not others, clearly showed two bands and were changed to the same degree, we quantified both bands. As an internal reference, rabbit anti-β-actin primary antibody (1:5000) was used. The values of each sample were normalized with the corresponding amount of β-actin. The ratio of phosphoprotein to total protein was described as phosphorylation level.

**Table 1:** Primary antibodies used in the present study.

Antigen	Host	Manufacturer (catalog number)	Dilution used
ERK1/2	Rabbit	Biorbyt (Orb160960)	1:2,000
GluR1	Mouse	Synaptic system (182011)	1:500
JNK	Rabbit	Protein tech (10023-1-AP)	1:1,000
Mitochondrial marker (Mitochondrial complex IV subunit 1, MTCO1)	Mouse	Abcam (#ab14705)	1:500
pERK1/2	Rabbit	Bioss (bs-3330R)	1:1,000
pGluR1-S831	Rabbit	Abcam (ab109464)	1:5,000
pGluR1-S845	Rabbit	Millipore (AB5849)	1:1,000
pJNK	Rabbit	Millipore (#07-105)	1:1,000
PKC	Rabbit	Abcam (ab23511)	1:1,000
PP1	Rabbit	Abcam (ab52619)	1:5,000
PP2A	Rabbit	Cell Signaling (#2038)	1:5,000
PP2B	Rabbit	Millipore (07-068-I)	1:1,000
pPKC	Rabbit	Abcam (ab59411)	1:1,000
pPP1	Rabbit	Abcam (ab62334)	1:5,000
pPP2A	Rabbit	Sigma (SAB4503975)	1:1,000
pPP2B	Rabbit	Badrilla (A010-80)	1:1,000
β-Actin	Mouse	Sigma (A5316)	1:5,000


### Immunohistochemistry and Measurement of Mitochondrial Length

Animals (*n* = 7 in each group, respectively) were anesthetized with urethane (1.5 g/kg, i.p.) and then transcardially perfused with 4% paraformaldehyde (pH 7.4). The brains were removed and post-fixed overnight in the same solution, then sequentially placed in 30% sucrose at 4°C. Coronal sections were cut at a thickness of 30 μm on a cryostat. During sections, we confirmed the intracerebroventricular location of a brain infusion kit. Free-floating sections were first incubated with 10% normal goat serum (Vector, Burlingame, CA, United States) in PBS for 30 min at room temperature. Sections were then incubated in a mitochondrial marker (Mitochondrial complex IV subunit 1, MTCO1, Abcam, United Kingdom) as the primary antibody (in PBS containing 0.3% Triton X-100) at room temperature overnight. After washing in PBS, sections were incubated for 1 h in a Cy3-conjugated secondary antiserum. For negative control, the hippocampal tissues were incubated with pre-immune serum instead of primary antibody. As the result of the negative control test, no immunoreactive structure was observed. Images were captured using an AxioImage M2 microscope or a confocal laser scanning microscope (LSM 710, Carl Zeiss, Inc., Oberkochen, Germany). Individual mitochondrion length in PV cells and CA1 neurons (*n* = 20/section) was measured by using ZEN lite software (Blue Edition, Carl Zeiss, Inc., Oberkochen, Germany) following 3D-reconstruction: based on our previous study (Kim and Kang, 2017; Ko and Kang, 2017), 25 serial images (z-stack, 1 μm) were obtained from each hippocampal section. Serial images were stacked, aligned, visualized and converted into 3D images using ZEN lite program. Thereafter, individual mitochondrial length (long axis) was measured. Two different investigators who were blind to the classification of tissues performed the measurement of mitochondrial length.

### Data Analysis

One-way ANOVA was used to determine statistical significance of data. For *post hoc* multiple comparisons, Bonferroni’s test was applied. A *p*-value below 0.05 was considered statistically significant.

## Results

### Effects of MK801 and Perampanel on Mitochondrial Dynamics

**FIGURE 1:**
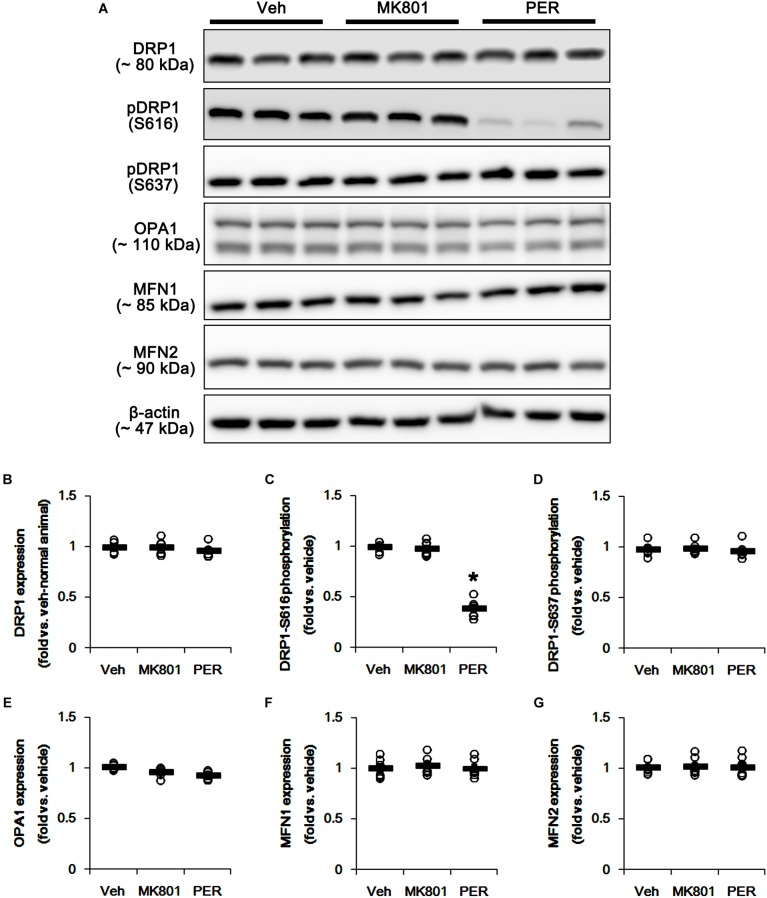
Effects of MK801 and perampanel (PER) on expressions and phosphorylations of mitochondrial dynamics-related molecules. Perampanel, but not MK801, reduces only DRP1-S616 phosphorylation level. **(A)** Representative images for western blot of DRP1, phospho (p)-DRP1-S616, pDRP1-637, OPA1, MFN1 and MFN2 in the hippocampal tissues. **(B–G)** Quantifications of DRP1, pDRP1-S616, pDRP1-637, OPA1, MFN1, and MFN2 levels. Open circles indicate each individual value. Horizontal bars indicate mean value. Error bars indicate SEM (*^∗^p* < 0.05 vs. vehicle; *n* = 7, respectively).

**FIGURE 2:**
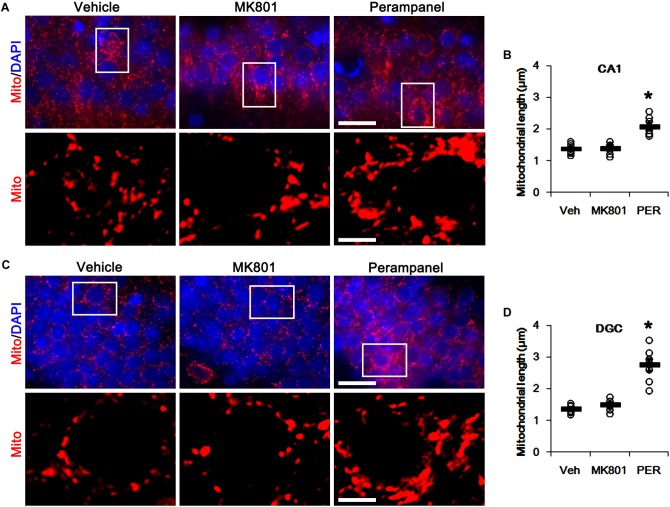
Effects of MK801 and perampanel (PER) on mitochondrial length. Perampanel elongates mitochondrial length in CA1 neurons **(A,B)** and DGC **(C,D)**, while MK801 does not. **(A)** Representative photos of mitochondria (Mito, red) and nuclei (DAPI, blue) in CA1 neurons. Bar = 25 (upper panels) and 5 (lower panels) μm. **(B)** Quantification of the mitochondrial length in CA1 neurons. Open circles indicate each individual value. Horizontal bars indicate mean value. Error bars indicate SEM (^∗^*p* < 0.05 vs. vehicle; *n* = 7, respectively). **(C)** Representative photos of mitochondria (Mito, red) and nuclei (DAPI, blue) in DGC neurons. Bar = 25 (upper panels) and 5 (lower panels) μm. **(D)** Quantification of the mitochondrial length in DGC. Open circles indicate each individual value. Horizontal bars indicate mean value. Error bars indicate SEM (^∗^*p* < 0.05 vs. vehicle; *n* = 7, respectively).

First, we investigated whether MK801 or perampanel affects expressions or phosphorylations of mitochondrial dynamics-related molecules. Figure 1 shows that both MK801 and perampanel did not influence DRP1, OPA1, and MFN1/2 expression levels. However, perampanel, but not MK801, reduced DRP1-S616 phosphorylation to 0.4-fold of vehicle level in the hippocampus (*p* < 0.05, *n* = 7, respectively; Figure 1A,C and Supplementary Figure 1), while both compounds could not affect its S637 phosphorylation level (Figure 1A,D). Since S616 phosphorylation increases DRP1 activity to induce mitochondrial fission (Campello and Scorrano, 2010; Kim and Kang, 2017), our findings indicate that the reduction in DRP-S616 phosphorylation induced by perampanel would result in mitochondrial elongation. Consistent with the diminished DRP1-S616 phosphorylation in western blot data, an immunofluorescent study revealed that perampanel elongated mitochondrial length in dentate granule cells (DGCs) as well as CA1 neurons (*p* < 0.05 vs. vehicle, *n* = 7, respectively; Figure 2A–D and Supplementary Figure 2), while MK801 did not. The average mitochondrial length in CA1 neurons was 1.39, 1.41, and 2.1 μm in vehicle-, MK801- and perampanel-treated animals, respectively. In DGCs, the average mitochondrial length was 1.37, 1.5, and 2.78 μm in vehicle-, MK801- and perampanel-treated animals, respectively. Therefore, these findings indicate that blockade of AMPAR, but not NMDAR, may facilitate mitochondrial fusion in the hippocampus under physiological condition.

### Effects of MK801 and Perampanel on ERK1/2, PKC, JNK, and p38 MAPK

**FIGURE 3:**
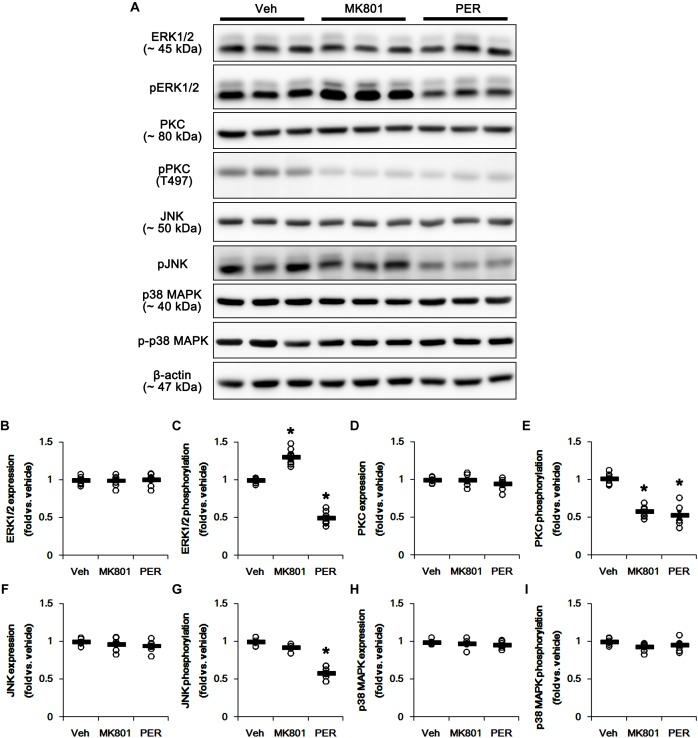
Effects of MK801 and perampanel (PER) on expressions and phosphorylations of ERK1/2, PKC, JNK, and p38 MAPK. MK801 increases ERK1/2 phosphorylation level, while perampanel reduces it. Both MK801 and perampanel reduce PKC phosphorylation. Perampanel, but not MK801, reduces JNK phosphorylation level. p38 MAPK expression and its phosphorylation are unaffected by MK801 and perampanel. **(A)** Representative images for western blot of ERK1/2, phospho (p)-ERK1/2, PKC, pPKC, JNK, pJNK, p38 MAPK, and p-p38 MAPK in the hippocampal tissues. **(B–I)** Quantifications of ERK1/2, pERK1/2, PKC, pPKC, JNK, pJNK, p38 MAPK, and p-p38 MAPK levels. Open circles indicate each individual value. Horizontal bars indicate mean value. Error bars indicate SEM (*^∗^p* < 0.05 vs. vehicle; *n* = 7, respectively).

The phosphorylation of DRP1 on S616 site activates DRP1-mediated mitochondrial fission, which is regulated by protein kinases such as extracellular-signal-regulated kinase 1/2 (ERK1/2; Prieto et al., 2016), protein kinase C (PKC; Lim et al., 2015), c-Jun N-terminal kinase (JNK; Peng et al., 2016), and p38 mitogen-activated protein kinase (p38 MAPK; Zhang et al., 2015). Thus, we investigated what kinds of protein kinases are involved in mitochondrial elongation induced by perampanel. Neither MK801 nor perampanel affected ERK1/2, PKC, JNK, and p38 MAPK expression levels (Figure 3A,B,D,F,H and Supplementary Figure 3). MK801 increased ERK1/2 phosphorylation level to 1.31-fold of vehicle level, while it reduced PKC phosphorylation level to 0.58-fold of vehicle level (*p* < 0.05 vs. vehicle, *n* = 7, respectively; Figure 3A,C,E and Supplementary Figure 3). Consistent with our previous study (Kim et al., 2019), perampanel diminished ERK1/2 and PKC phosphorylation levels to 0.5- and 0.53-fold of vehicle levels, respectively (*p* < 0.05 vs. vehicle, *n* = 7, respectively; Figure 3A,C,E and Supplementary Figure 3). In addition, perampanel decreased JNK phosphorylation to 0.58-fold of vehicle level (*p* < 0.05 vs. vehicle, *n* = 7, respectively; Figure 3A,G and Supplementary Figure 3), while MK801 did not influence it (Figure 3A,G and Supplementary Figure 3). p38 MAPK phosphorylation level was unaltered by both MK801 and perampanel (Figure 3A,I and Supplementary Figure 3). These findings indicate that the distinct effects of MK801 and perampanel on ERK1/2 and JNK activities may differently affect mitochondrial dynamics under physiological condition.

### Effects of MK801 and Perampanel on Protein Phosphatases

**FIGURE 4:**
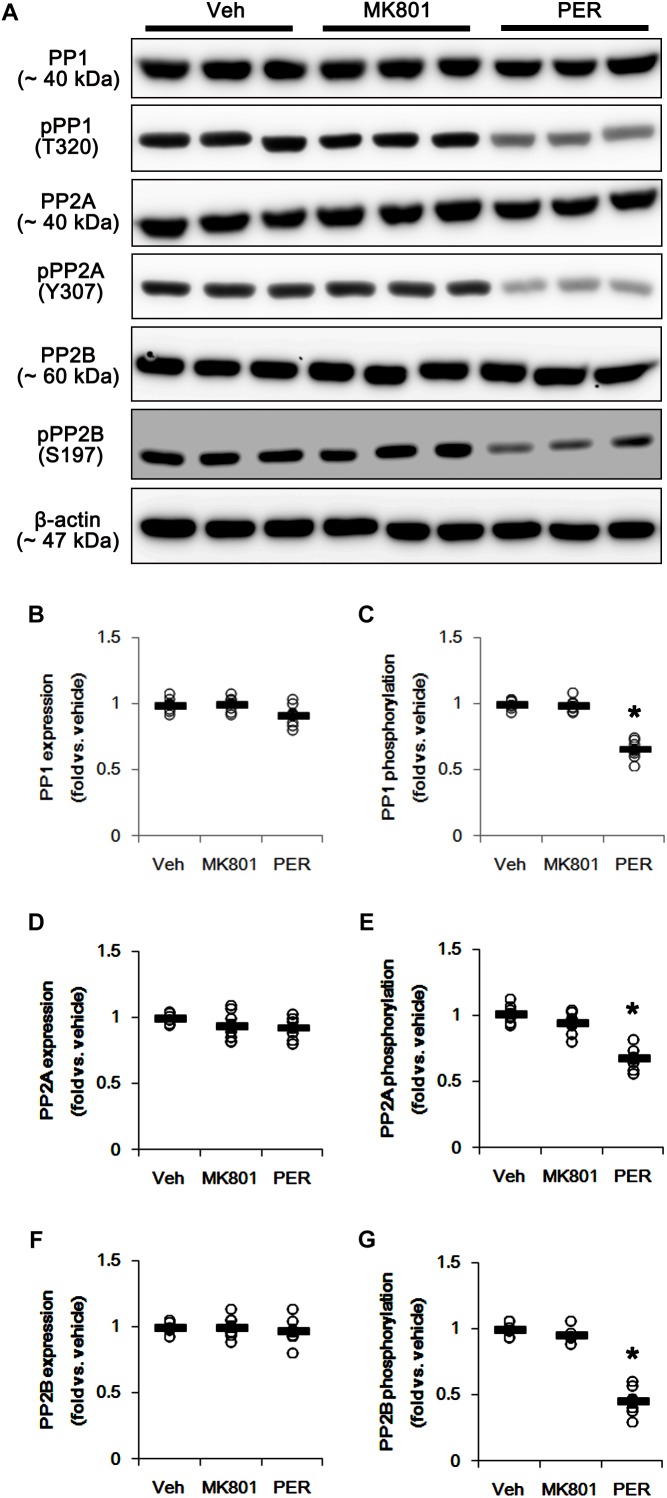
Effects of MK801 and perampanel (PER) on expressions and phosphorylations of PP1, PP2A, and PP2B. Perampanel reduces PP1, PP2A, and PP2B phosphorylations, while MK801 does not. **(A)** Representative images for western blot of PP1, phospho (p)-PP1, PP2A, pPP2A, PP2B, and pPP2B in the hippocampal tissues. **(B–G)** Quantifications of PP1, pPP1, PP2A, pPP2A, PP2B, and pPP2B levels. Open circles indicate each individual value. Horizontal bars indicate mean value. Error bars indicate SEM (*^∗^p* < 0.05 vs. vehicle; *n* = 7, respectively).

Since DRP1 activity is also regulated by various protein phosphatase activities (Campello and Scorrano, 2010), protein phosphatases (PPs) would be involved in the different effects of MK801 and perampanel on mitochondrial dynamics. Thus, we tested whether MK801 and perampanel affect the activities of PPs in the hippocampus. MK801 did not influence the expression and phosphorylation of PP1, PP2A, and PP2B (Figure 4A–G and Supplementary Figure 4). Consistent with our previous study (Kim et al., 2019), perampanel reduced PP1, PP2A, and PP2B phosphorylations to 0.66-, 0.6-, and 0.46-fold of vehicle levels, although it did not affect their expression levels (*p* < 0.05 vs. vehicle, *n* = 7, respectively; Figure 4A–G and Supplementary Figure 4). Since the phosphorylations inhibit the activities of protein phosphates (Hashimoto et al., 1988; MacDonnell et al., 2009), our findings indicate that perampanel, but not MK801, may activate PP1, PP2A, and PP2B in the rat hippocampus.

### Effects of Various Inhibitors of Kinases and PPs on Mitochondrial Dynamics

**FIGURE 5:**
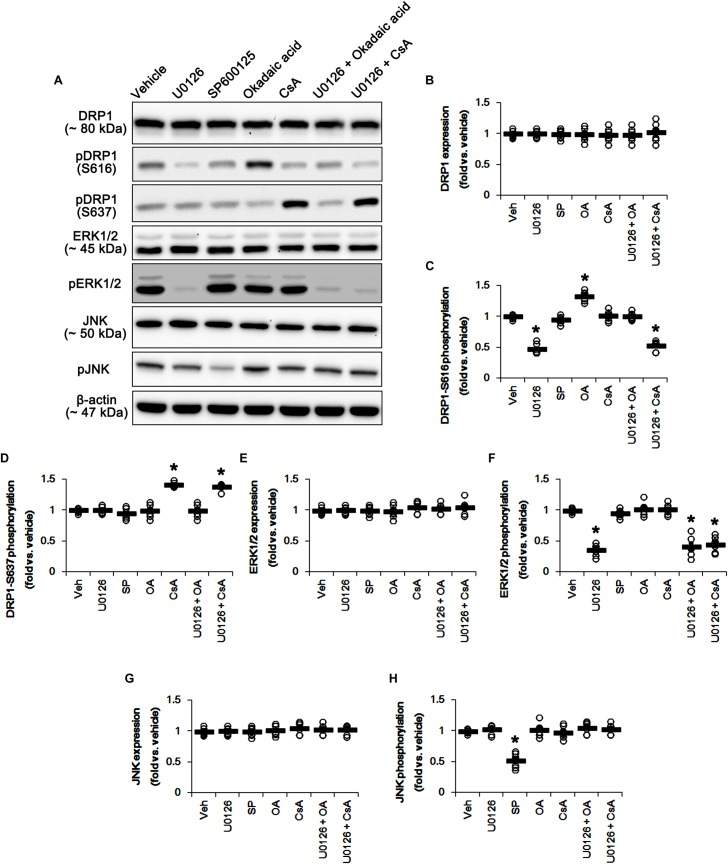
Effects of U0126, SP600125 (SP), okadaic acid (OA), CsA, U0126 + OA and U0126 + CsA on expressions and phosphorylations of DRP1, ERK1/2, and JNK. U0126 reduces DRP1-S616, not -S637, phosphorylation level. SP600125 does not influence DRP1-S616 and -S637 phosphorylations. Okadaic acid and CsA increase DRP1-S616 and -S637 phosphorylation, respectively. **(A)** Representative images for western blot of DRP1, phospho (p)-DRP1-S616, pDRP1-S637, ERK1/2, pERK1/2, JNK, and pJNK in the hippocampal tissues. **(B–H)** Quantifications of DRP1, pDRP1-S616, pDRP1-S637, ERK1/2, pERK1/2, JNK, and pJNK levels. Open circles indicate each individual value. Horizontal bars indicate mean value. Error bars indicate SEM (*^∗^p* < 0.05 vs. vehicle; *n* = 7, respectively).

Based on the present data aforementioned, we validated the effects of U0126 (an ERK1/2 inhibitor), SP600125 (a JNK inhibitor), okadaic acid (a PP1/PP2A inhibitor) and CsA (a PP2B inhibitor) on DRP1 phosphorylation and mitochondrial length to confirm the underlying mechanism of perampanel for mitochondrial dynamics. All chemicals did not affect DRP1 expression in the hippocampus (Figure 5A,B and Supplementary Figure 5). However, U0126 reduced DRP1-S616, not -S637, phosphorylation (*p* < 0.05 vs. vehicle, *n* = 7, respectively; Figure 5A,C,D and Supplementary Figure 5). Okadaic acid and CsA increased DRP1-S616 and -S637 phosphorylation, respectively (*p* < 0.05 vs. vehicle, *n* = 7, respectively; Figure 5A,C,D and Supplementary Figure 5). SP600125 did not influence DRP1-S616 and -S637 phosphorylations (Figure 5A,C,D and Supplementary Figure 3). To confirm the cooperation of ERK1/2 inhibition and PPs activations for the regulation of DRP1 phosphorylation, we applied co-treatment of U0126 + okadaic acid or + CsA. Co-treatment of okadaic acid abrogated U0126-induced reduction in DRP1-S616 phosphorylation (*p* < 0.05 vs. vehicle, *n* = 7, respectively; Figure 5A,C,D and Supplementary Figure 5), while co-treatment of CsA did not affect it. These findings indicate that both ERK1/2 and PP1/PP2A may coordinately regulate DRP1-S616 phosphorylation.

**FIGURE 6:**
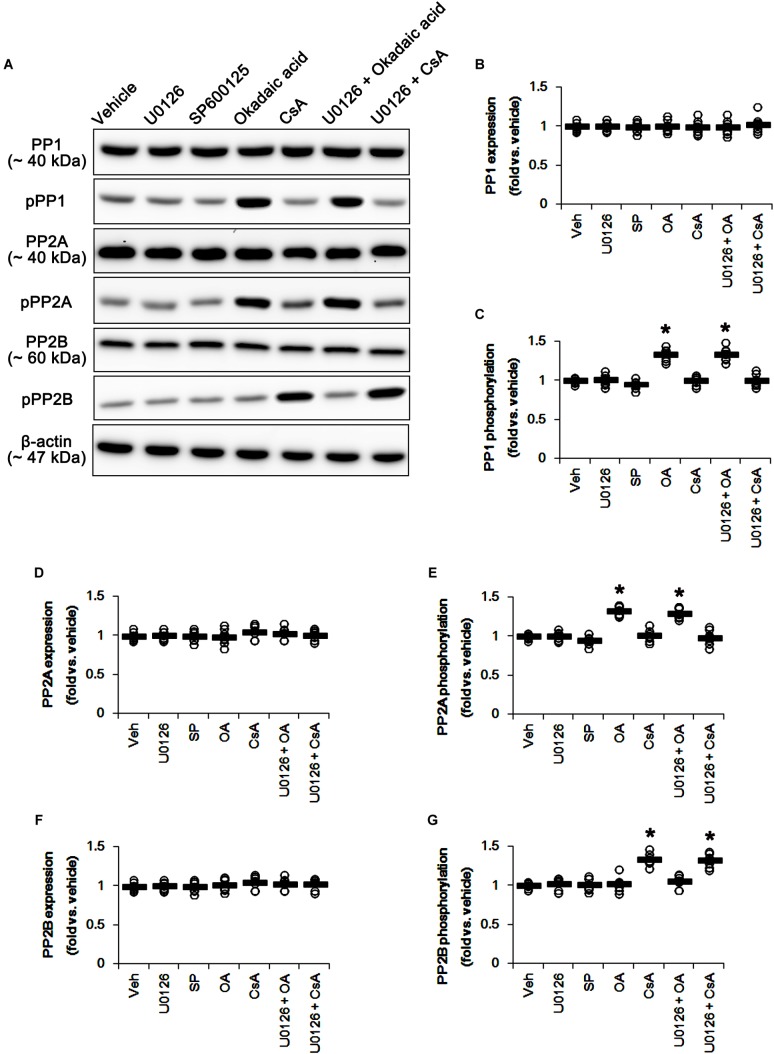
Effects of U0126, SP600125 (SP), okadaic acid (OA), CsA, U0126 + OA and U0126 + CsA on expressions and phosphorylations of PP1, PP2A, and PP2B. Okadaic acid and CsA increase PP1/PP2A and PP2B phosphorylations, respectively. **(A)** Representative images for western blot of PP1, phospho (p)-PP1, PP2A, pPP2A, PP2B, and pPP2B in the hippocampal tissues. **(B–G)** Quantifications of PP1, pPP1, PP2A, pPP2A, PP2B, and pPP2B levels. Open circles indicate each individual value. Horizontal bars indicate mean value. Error bars indicate SEM (*^∗^p* < 0.05 vs. vehicle; *n* = 7, respectively).

U0126 and SP600152 reduced ERK1/2 and JNK phosphorylations without altering their expression levels (*p* < 0.05 vs. vehicle, *n* = 7, respectively; Figure 5A,E–H and Supplementary Figure 5). Consistent with previous studies (Liu et al., 2008; Hou et al., 2013), okadaic acid and CsA also enhanced increased PP1/PP2A and PP2B phosphorylation levels without affecting their expression levels, respectively (*p* < 0.05 vs. vehicle, *n* = 7, respectively; Figure 6A–G and Supplementary Figure 6). In addition, co-treatment of U0126 did not influence the effects of okadaic acid and CsA on PP phosphorylations (Figure 6A–G and Supplementary Figure 6). These findings also suggest that each inhibitor may not show off-target effects in the present study.

**FIGURE 7:**
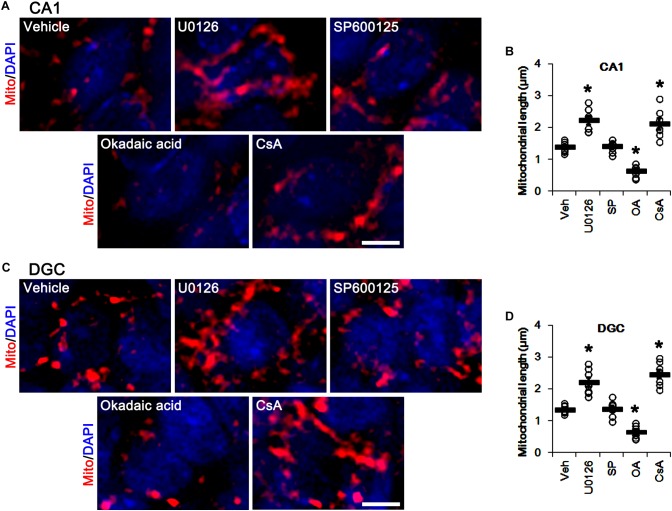
Effects of U0126, SP600125 (SP), okadaic acid (OA) and CsA on mitochondrial length. U0126 and CsA elongate mitochondrial length in CA1 neurons **(A,B)** and DGCs **(C,D)**, while okadaic acid decreases it in both neurons. SP600125 does not affect mitochondrial length. **(A)** Representative photos of mitochondria (Mito, red) and nuclei (DAPI, blue) in CA1 neurons. Bar = 5 μm. **(B)** Quantification of the mitochondrial length in CA1 neurons. Open circles indicate each individual value. Horizontal bars indicate mean value. Error bars indicate SEM (^∗^*p* < 0.05 vs. vehicle; *n* = 7, respectively). **(C)** Representative photos of mitochondria (Mito, red) and nuclei (DAPI, blue) in DGC neurons. Bar = 5 μm. **(D)** Quantification of the mitochondrial length in DGC. Open circles indicate each individual value. Horizontal bars indicate mean value. Error bars indicate SEM (^∗^*p* < 0.05 vs. vehicle; *n* = 7, respectively).

Similar to DRP1 phosphorylations, U0126 and CsA elongated mitochondrial length in CA1 neurons and DGCs (*p* < 0.05 vs. vehicle, *n* = 7, respectively; Figure 7A–D), while okadaic acid resulted in mitochondrial fragmentation in both neuronal subpopulations (*p* < 0.05 vs. vehicle, *n* = 7, respectively; Figure 7A–D). SP600125 did not affect mitochondrial length, as compared to vehicle (Figure 7A–D). Together with the data obtained from perampanel treatment, our findings suggest that blockade of AMPAR induced by perampanel may elongate mitochondrial length via inhibiting ERK1/2 activity as well as enhancing PP1/PP2A activities.

### Effects of Okadaic Acid and CsA on DRP1, ERK1/2, JNK, and PP Phosphorylations Induced by Perampanel

**FIGURE 8:**
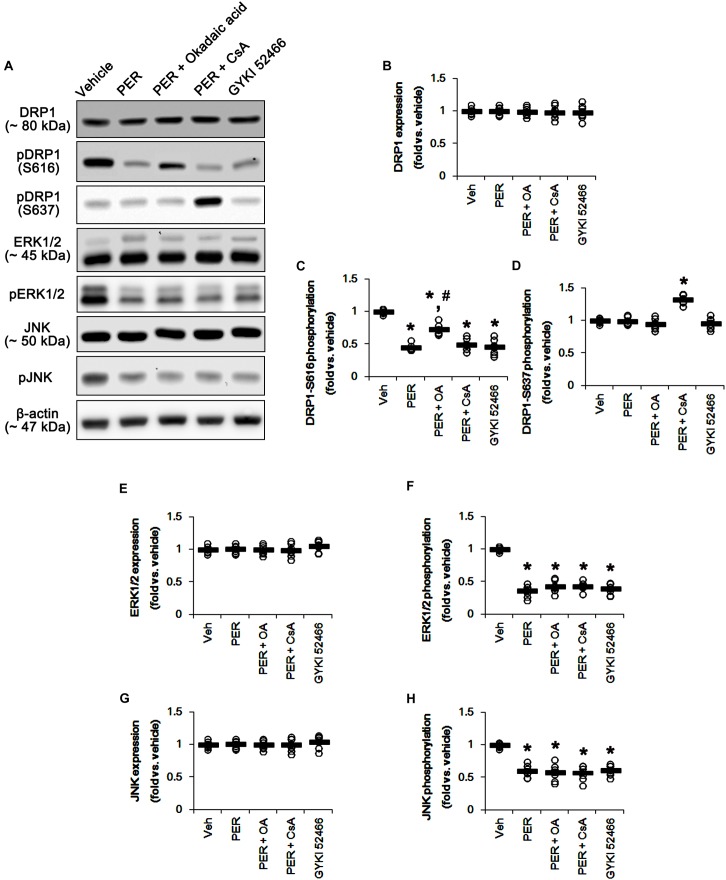
Effects of co-treatment of perampanel (PER) with okadaic acid (OA) or CsA, and GYKI 52466 on expressions and phosphorylations of DRP1, ERK1/2, and JNK. Co-treatment of okadaic acid and CsA with perampanel significantly attenuates the reductions in DRP1-S616 and -S637 phosphorylations without changing DRP1 expression level, but not ERK1/2 and JNK phosphorylations, induced by perampanel. Similar to perampanel, GYKI 52466 reduces DRP1-S616 phosphorylation, but not S637 phosphorylation level, without affecting DRP1 expression. GYKI 52466 also abolishes ERK1/2 and JNK phosphorylation levels. **(A)** Representative images for western blot of DRP1, phospho (p)-DRP1-S616, pDRP1-S637, ERK1/2, pERK1/2, JNK, and pJNK levels in the hippocampal tissues. **(B–H)** Quantifications of DRP1, pDRP1-S616, pDRP1-S637, ERK1/2, pERK1/2, JNK, and pJNK levels. Open circles indicate each individual value. Horizontal bars indicate mean value. Error bars indicate SEM (^∗^, ^#^*p* < 0.05 vs. vehicle and PER-treated animals; *n* = 7, respectively).

**FIGURE 9:**
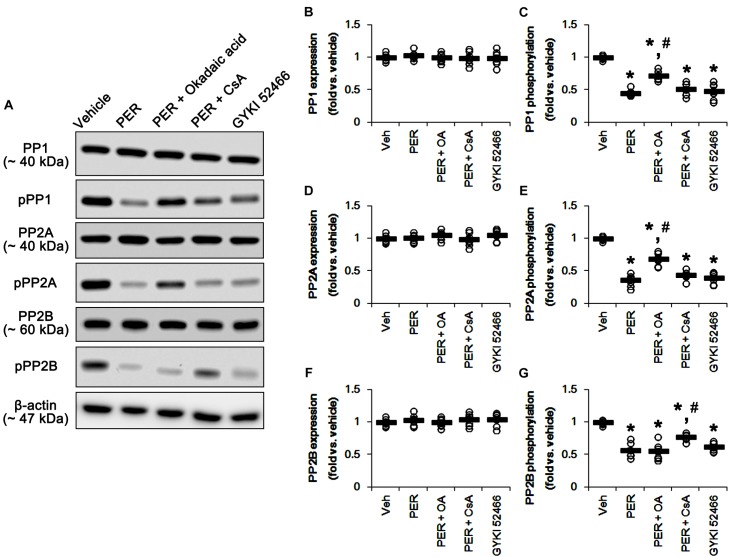
Effects of co-treatment of perampanel (PER) with okadaic acid (OA) or CsA, and GYKI 52466 on expressions and phosphorylations of PP1, PP2A, and PP2B. Co-treatment of okadaic acid and CsA significantly alleviates the reductions in PP1/PP2A and PP2B phosphorylations induced by perampanel without altering PPs expression levels, respectively. GYKI 52466 decreases PP1, PP2A, and PP2B phosphorylations. **(A)** Representative images for western blot of PP1, phospho (p)-PP1, PP2A, pPP2A, PP2B, and pPP2B levels in the hippocampal tissues. **(B–G)** Quantifications of PP1, pPP1, PP2A, pPP2A, PP2B, and pPP2B levels. Open circles indicate each individual value. Horizontal bars indicate mean value. Error bars indicate SEM (^∗^, ^#^*p* < 0.05 vs. vehicle and PER-treated animals; *n* = 7, respectively).

Since perampanel effectively activated (reduced phosphorylation level) PP1/PP2A and PP2B, we also applied co-treatment of okadaic acid, or CsA, with perampanel to investigate the role of PPs on DRP1 phosphorylation induced by perampanel. Co-treatment of okadaic acid and CsA with perampanel significantly attenuated the reductions in DRP1-S616 and -S637 phosphorylations without changing DRP1 expression level, respectively (*p* < 0.05 vs. vehicle and perampanel, respectively; *n* = 7, respectively; Figure 8A–D and Supplementary Figure 7). Co-treatments of okadaic acid and CsA did not affect the reductions in ERK1/2 and JNK phosphorylations induced by perampanel (Figure 8E–H and Supplementary Figure 7). In addition, co-treatment of okadaic acid and CsA significantly alleviated the reductions in PP1/PP2A and PP2B phosphorylations without altering PPs expression levels, respectively (*p* < 0.05 vs. vehicle, *n* = 7, respectively; Figure 9A–G and Supplementary Figure 8). These findings indicate that perampanel-mediated PP1/PP2A activation may elongate mitochondrial length via DRP1-S616 dephosphorylation.

### Effects of GYKI 52466 on DRP1, ERK1/2, JNK, and PP Phosphorylations

To confirm the role of blockade of AMPAR in DRP1 phosphorylations, we also investigated the effects of GYKI 52466,another allosteric AMPAR inhibitor (a non-competitive AMPAR antagonist), on DRP phosphorylations and activities of kinases as well as PPs. Similarly to perampanel, GYKI 52466 reduced DRP1-S616 phosphorylation but not S637 phosphorylation level, without affecting DRP1 expression (*p* < 0.05 vs. vehicle, *n* = 7, respectively; Figure 8A–D and Supplementary Figure 7). GYKI 52466 also abolished ERK1/2 and JNK phosphorylation levels (*p* < 0.05 vs. vehicle, *n* = 7, respectively; Figure 8A,E–H and Supplementary Figure 7). In addition, GYKI 52466 decreased PP1, PP2A, and PP2B phosphorylations (*p* < 0.05 vs. vehicle, *n* = 7, respectively; Figure 9A–G and Supplementary Figure 8). Therefore, our findings indicate that allosteric AMPAR inhibition may commonly affect ERK1/2, JNK, PP1, PP2A, and PP2B activities in the normal rat hippocampus.

### Effects of MK801 and Perampanel on GluR1 Phosphorylation

**FIGURE 10:**
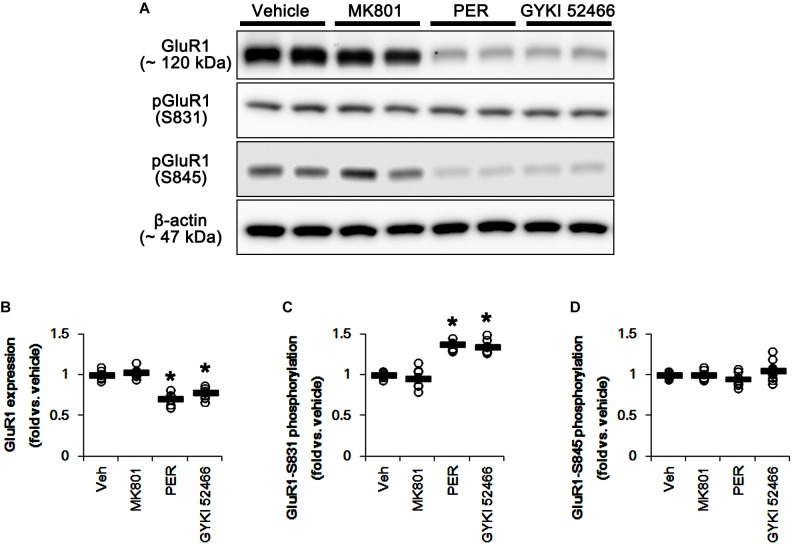
Effects of MK801, perampanel (PER) and GYKI 52466 on GluR1 expression and its phosphorylations. MK801 does not affect GluR1 expression and its phosphorylations. Perampanel and GYKI 52466 reduce GluR1 expression, but increase GluR1-S831, not -S845, phosphorylation. **(A)** Representative images for western blot of GluR1, phospho (p)-GluR1-S831 and pGluR1-S845 levels in the hippocampal tissues. **(B–D)** Quantifications of GluR1, pGluR1-S831, and pGluR1-S845 levels. Open circles indicate each individual value. Horizontal bars indicate mean value. Error bars indicate SEM (^∗^*p* < 0.05 vs. vehicle; *n* = 7, respectively).

*N*-Methyl-D-aspartate receptor activity affects AMPAR functionality. Briefly, GluR1, a subunit of AMPAR, is phosphorylated at serine (S) 831 site when NMDAR activation, while GluR1-S845 phosphorylation was decreased (Ai et al., 2011). Therefore, the effects of MK801 and perampanel on AMPAR phosphorylations are noteworthy, which would lead to the distinct mitochondrial dynamics. In the present study, MK801 did not affect GluR1 expression and its S831 and S845 phosphorylations (Figure 10 and Supplementary Figure 9). In contrast to MK801, perampanel, and GYKI 52466 reduced GluR1 expression to 0.7- and 0.77-fold of vehicle level, respectively (*p* < 0.05 vs. vehicle, *n* = 7, respectively; Figure 10A,B and Supplementary Figure 9). However, GluR1-S831 phosphorylation was increased to 1.38- and 1.35-fold of vehicle level, respectively (*p* < 0.05 vs. vehicle, *n* = 7, respectively; Figure 10A,C and Supplementary Figure 9). GluR1-S845 phosphorylation was unaffected by MK801, perampanel or GYKI 52466 (Figure 10A,D and Supplementary Figure 9). These findings indicate that MK801 may not affect AMPAR activity, and that the selective AMPA inhibition by perampanel or GYKI 52466 may evoke mitochondrial elongation under physiological condition.

## Discussion

The major findings in the present study are that blockade of AMPAR elongated mitochondrial length by regulating ERK1/2- and PP1/PP2A-mediated DRP1-S616 phosphorylations under physiological conditions.

*N*-Methyl-D-aspartate receptor- and AMPAR-mediated intracellular Ca^2+^ overloads evoke necrosis or delayed apoptosis of neurons via bioenergetic collapse, activation of calpains, oxidative stress, and mitochondrial dysfunctions (Choi, 1992; Arundine and Tymianski, 2004; Li and Wang, 2016). The impaired mitochondrial dynamics also contribute to neuronal death in various neurological diseases (Rintoul et al., 2003; Grohm et al., 2012; Kim et al., 2014; Ruiz et al., 2018). Interestingly, the roles of excessive mitochondrial fission in neuronal death show a receptor specific manner, since Mdivi-1 attenuates mitochondrial fission induced by NMDA, but not AMPA (Ruiz et al., 2018). However, it is still unknown whether NMDAR or AMPAR activity contributes to mitochondrial dynamics under physiological conditions. In the present study, MK801 increased ERK1/2 phosphorylation, but reduced PKC phosphorylation. These findings are consistent with previous reports demonstrating the effect of MK801 on ERK1/2, JNK, and p38 MAPK (Chen et al., 2003; Seo et al., 2007). However, we found that MK801 did not affect mitochondrial length in both CA1 neurons and DGCs as well as phosphorylation levels of DRP1, JNK, and p38 MAPK. Thus, these findings indicate that blockade of NMDAR may not contribute to mitochondrial dynamics under physiological condition.

Unlike MK801, the present data demonstrate that perampanel elongated mitochondrial length in both CA1 neurons and DGCs. Furthermore, perampanel and GYKI 52466 reduced DRP1-S616 phosphorylation accompanied by diminishing phosphorylations (activations) of ERK1/2, PKC and JNK, but not p38 MAPK. Based on these results, it is likely that the blockade of AMPAR would inhibit mitochondrial fission by decreasing ERK1/2 and JNK activities. However, our other studies reveal that U0126 (an ERK1/2 inhibitor), but not SP600125 (a JNK inhibitor), abrogated DRP1-S616 phosphorylation and evoked mitochondrial elongation. Since AMPAR activation increases ERK1/2 phosphorylation (Mao et al., 2004), therefore, our findings suggest that AMPAR inhibition may lead to mitochondrial elongation by inhibiting ERK1/2 activity.

Protein phosphatase activities also contribute to mitochondrial dynamics via dephosphorylating DRP1-S616 or -S637 site (Cereghetti et al., 2008; Cho et al., 2012; Park et al., 2015). DRP1-S616 dephosphorylation promotes mitochondrial fusion (Taguchi et al., 2007; Liesa et al., 2009), while DRP1-S637 dephosphorylation leads to the attachment of DRP1 to mitochondria and subsequently facilitate mitochondrial fission (Kashatus et al., 2011; Wang et al., 2012). Indeed, PP1/PP2A inhibition by okadaic acid results in mitochondrial fission by enhancing DRP1-S616 phosphorylation (Cho et al., 2012). In contrast, PP2B inhibition by CsA facilitates mitochondrial fusion with increasing DRP1-S637 phosphorylation (Cereghetti et al., 2008; Park et al., 2015). Consistent with these previous reports, the present study shows that okadaic acid and CsA increased DRP1-S616 and -S637 phosphorylation, respectively. In addition, CsA elongated mitochondrial length in CA1 neurons and DGCs, while okadaic acid provoked mitochondrial fragmentation in both neurons. Furthermore, in the present study, perampanel reduced PP1, PP2A, and PP2B phosphorylations indicating the activation of these PPs, which were unaffected by MK801. However, both MK801 and perampanel did not influence DRP1-S637 phosphorylation, while only perampanel declined DRP1-S616 phosphorylation. Together with the results obtained from PP inhibitor-treatments, our findings suggest that perampanel-induced PP1/PP2A activations (dephosphorylation) may also be involved in mitochondrial elongation via DRP1-S616 dephosphorylation.

In the present study, co-treatment of okadaic acid, but not CsA, abrogated U0126-induced reduction in DRP1-S616 phosphorylation, although co-treatment of U0126 did not influence the effects of okadaic acid and CsA on PP phosphorylations. Furthermore, co-treatments of okadaic acid and CsA significantly alleviated the reductions in PP1/PP2A and PP2B phosphorylations induced by perampanel, respectively. Co-treatment of okadaic acid and CsA with perampanel also attenuated the reductions in DRP1-S616 and -S637 phosphorylations without changing DRP1 expression level, respectively. However, co-treatments of okadaic acid and CsA did not affect perampanel-mediated reductions in ERK1/2 and JNK phosphorylations. These findings indicate that both ERK1/2 and PP1/PP2A may coordinately regulate DRP1-S616 phosphorylation, and that each inhibitor may not show off-target effects in the present study. Thus, our findings suggest that blockade of AMPAR may regulate the cooperation of ERK1/2- and PP1/PP2A for the modulation of DRP1 phosphorylations.

It is well-known that the phosphorylations of GluR1 subunit of AMPAR at S831 and S845 sites increase the conductance of AMPAR and potentiate rapid excitatory neurotransmission (Derkach et al., 1999; Snyder et al., 2000). Interestingly, AMPAR activity is distinctly regulated by NMDAR functionality: NMDAR activation phosphorylates GluR1 subunit at S831 site, but decreases GluR1-S845 phosphorylation (Ai et al., 2011). Therefore, it is considerable that the discrepancies of the effects of MK801 and perampanel on mitochondrial dynamics would be relevant to an indirect regulation of NMDAR to GluR1 phosphorylation. Consistent with a previous study (Zhang et al., 2014), the present study reveals that MK801 did not affect GluR1 expression and its phosphorylations. However, perampanel decreased GluR1 expression, while it increased GluR1-S831 phosphorylation ratio with the unaltered GluR1-S845 phosphorylation ratio. Since GluR1 phosphorylations represent the enhanced AMPAR-mediated synaptic currents (Zhang et al., 2016), our findings indicate that the increase in S831 phosphorylation ratio of GluR1 may be one of the adaptive responses for the reductions in AMPAR functionality or GluR1 expression level by perampanel. These findings also support that AMPAR rather than NMDAR may regulate mitochondrial dynamics under physiological condition.

The molecular machinery of mitochondrial dynamics is also regulated by MFN1/2 and OPA1, which elongate mitochondrial length (Westermann, 2008). Recently, it has been reported that BGP-15, a hydroxylamine derivative, promotes mitochondrial fusion by activating OPA1 and MFN1/2, accompanied by increasing ERK1/2 activity, but reducing JNK phosphorylation (Szabo et al., 2018). Post-myocardial infarction rat models also exhibit the reduced mitochondrial fusion, which is relevant to ERK1/2 and JNK activation and the reduced OPA1 and MFN2 expressions (Jiang et al., 2014). Furthermore, the inhibition of PP2A activity up-regulates DRP1, MFN1/2, and OPA1 protein levels (Plácido et al., 2017). In the present study, neither perampanel nor MK801 affected expression levels of MFN1/2 and OPA1. Thus, it is likely that glutamate-mediated neuronal excitability may influence only DRP1-, but not MFN1/2- or OPA1-, dependent mitochondrial dynamics. However, ERK1/2-mediated MFN1 phosphorylation leads to mitochondrial fusion (Pyakurel et al., 2015). In addition, PTEN-induced putative kinase protein 1 (PINK1) phosphorylates MFN2 and promotes its Parkin-mediated ubiqitination, which affects mitochondrial dynamics (Chen and Dorn, 2013). Thus, the possibility that perampanel would also affect OPA1 and MFN1/2 activity via certain phosphorylation events remains elusive and cannot be excluded.

On the other hand, the effects of AMPAR antagonist on GluR1 expression have been controversial. NBQX, a competitive AMPAR antagonist, increases GluR1 expression (Thiagarajan et al., 2005) or not (Maeng et al., 2008). Similarly to our recent study (Kim et al., 2019), both perampanel and GYKI 52466 down-regulated GluR1 expression, but increased GluR1-S831 phosphorylation. Although we could not explain the underlying mechanisms concerning these phenomena, it is plausible that the properties of non-competitive (allosteric) AMPAR antagonists, such as peramapenl and GYKI 52466, would result in the distinct effect on GluR1 expression as compared to competitive AMPAR antagonists. In addition, the serine residue phosphorylation plays an important role in regulating the conductance and trafficking of AMPAR (Lee et al., 1998, 2000; Shepherd and Huganir, 2007). Therefore, our findings suggest that the elevated GluR1-S831 phosphorylation by perampanel and GYKI 52466 may be one of the adaptive responses for the diminished GluR1 expression or AMPAR-mediated currents. This is because AMPAR activity in response to AMPA is regulated in negative feedback manners (Snyder et al., 2000). Further studies are needed to elucidate the regulatory mechanisms of GluR1 expression by allosteric AMPAR antagonists.

## Conclusion

To the best of our knowledge, the present data demonstrate the previously unreported AMPAR-mediated underlying mechanism for mitochondrial dynamics under physiological condition. Furthermore, our findings suggest that the regulation of AMPAR functionality may be one of the therapeutic targets for neurological diseases related to aberrant mitochondrial dynamics.

## Ethics Statement

Animal protocols were approved by the Institutional Animal Care and Use Committee of Hallym University (Chunchon, South Korea).

## Author Contributions

T-CK designed and supervised the project. J-EK, H-CC, H-KS, and T-CK performed the experiments described in the manuscript and analyzed the data. J-EK and T-CK wrote the manuscript.

## Conflict of Interest Statement

The authors declare that the research was conducted in the absence of any commercial or financial relationships that could be construed as a potential conflict of interest.
