# Supplementary material for: Blockade of AMPA Receptor Regulates Mitochondrial Dynamics by Modulating ERK1/2 and PP1/PP2A-Mediated DRP1-S616 Phosphorylations in the Normal Rat Hippocampus
Source: Front Cell Neurosci. 2019 May 1;13:179. doi: 10.3389/fncel.2019.00179 (PMC6504797; doi:10.3389/fncel.2019.00179)
Supplement: Supplementary file 1 [file Data_Sheet_1.PDF]

Supporting information

Blockade of AMPA receptor regulates mitochondrial dynamics by modulating ERK1/2 and PP1/PP2A-mediated DRP1-S616 phosphorylations in the normal rat hippocampus

Ji-Eun Kim[1,3], Hui-Chul Choi[2,3], Hong-Ki Song[2,3], Tae-Cheon Kang[1,3*]

[1]Department of Anatomy and Neurobiology, College of Medicine, Hallym University, Chunchon 24252, South Korea

[2]Department of Neurology, College of Medicine, Hallym University, Chunchon 24252, South Korea

[3]Institute of Epilepsy Research, College of Medicine, Hallym University, Chunchon 24252, South Korea

* Correspondence to: T. -C. Kang, Department of Anatomy and Neurobiology, College of Medicine, Hallym University, Chunchon, Kangwon-Do 24252, South Korea; Tel: +82-33-248-2524; Fax: +82-33-248-2525; E-mail: tckang@hallym.ac.kr

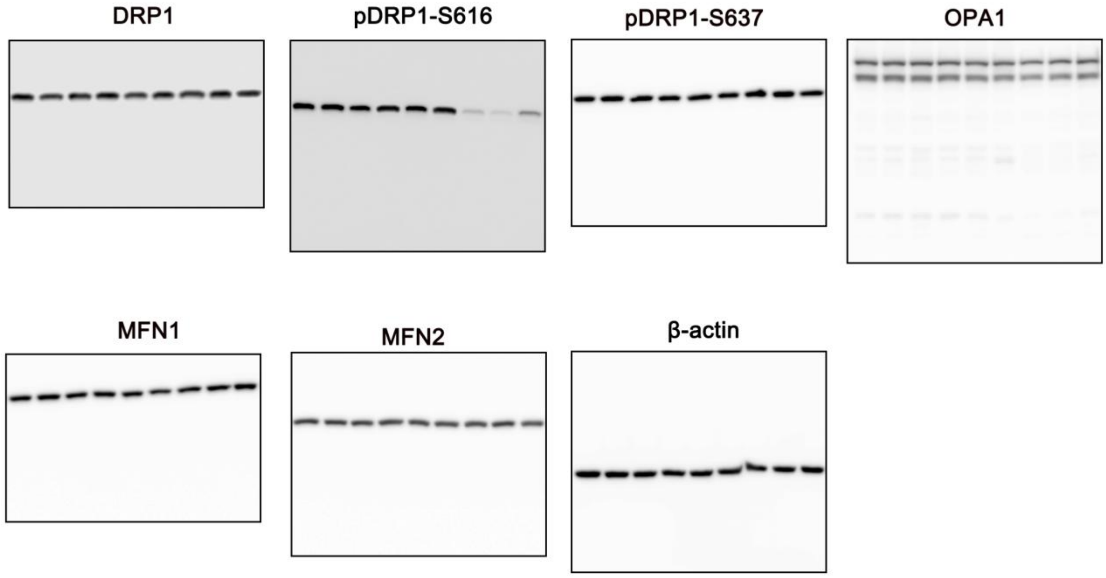

Supplementary Fig. 1. Full-length gel images of western blot data in Fig. 1.

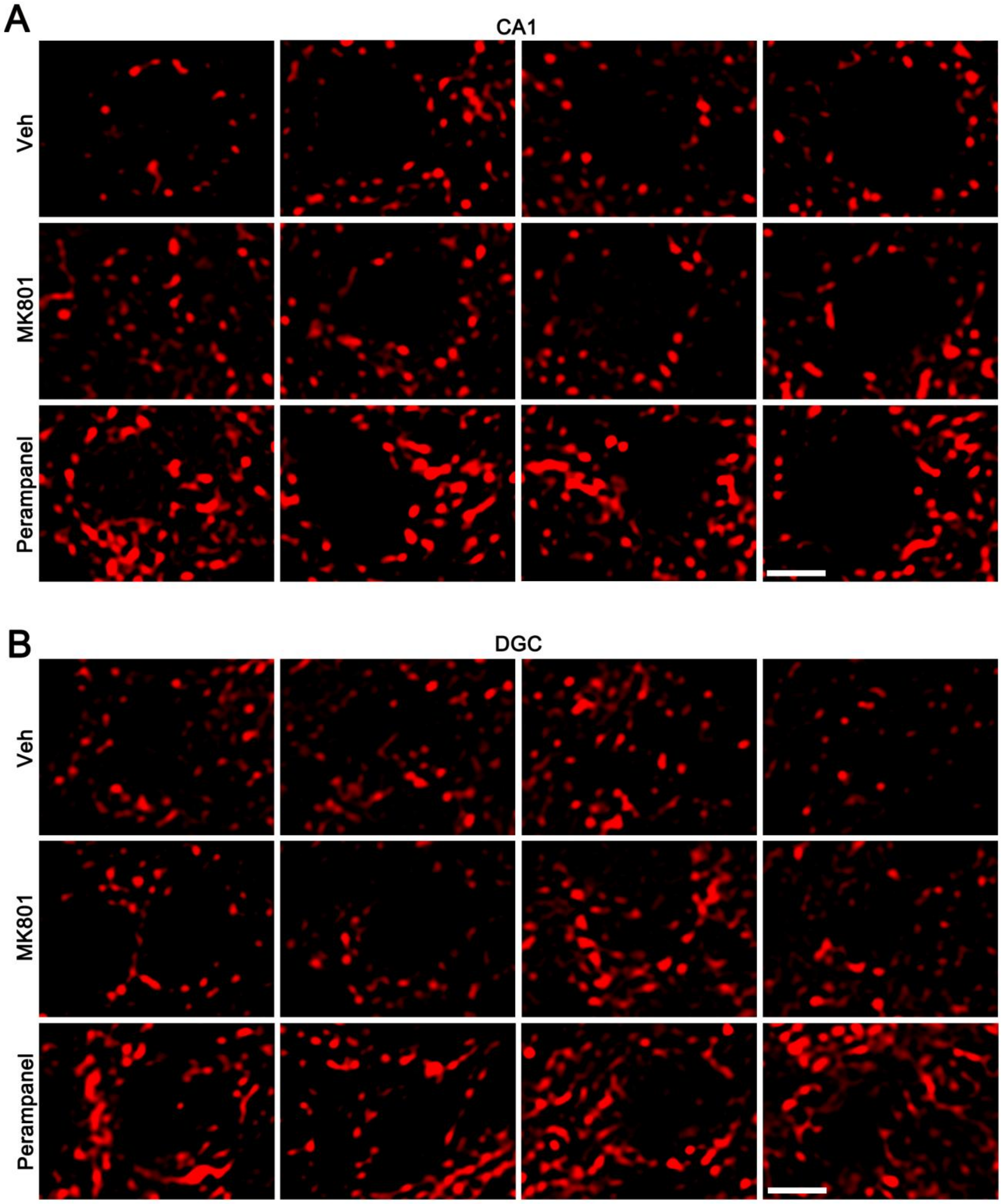

Supplementary Fig. 2. Representative images for analysis of mitochondrial length in Fig. 2 demonstrating effects of MK801 and perampanel (PER) on mitochondrial dynamics, which are obtained from each four animals. Bar = 5 μm.

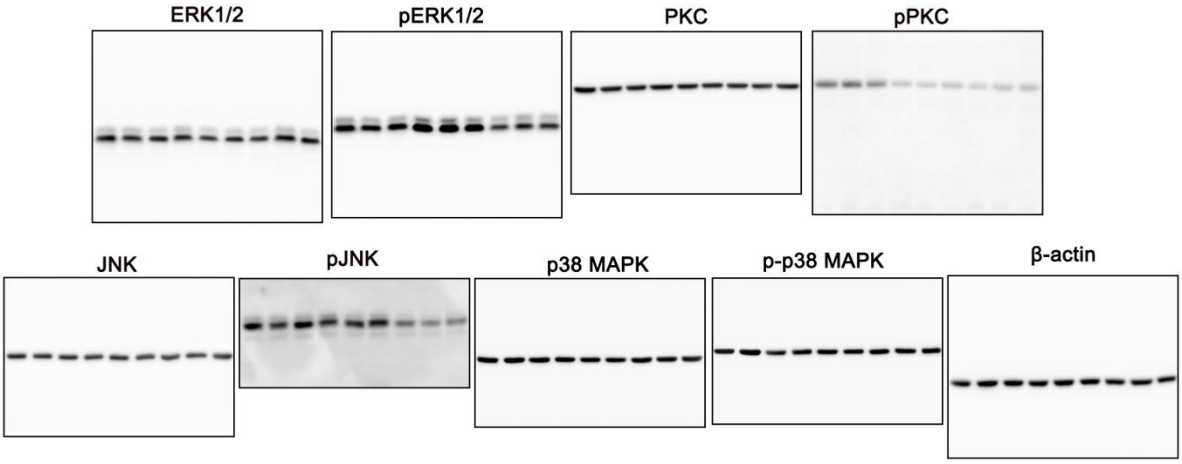

Supplementary Fig. 3. Full-length gel images of western blot data in Fig. 3.

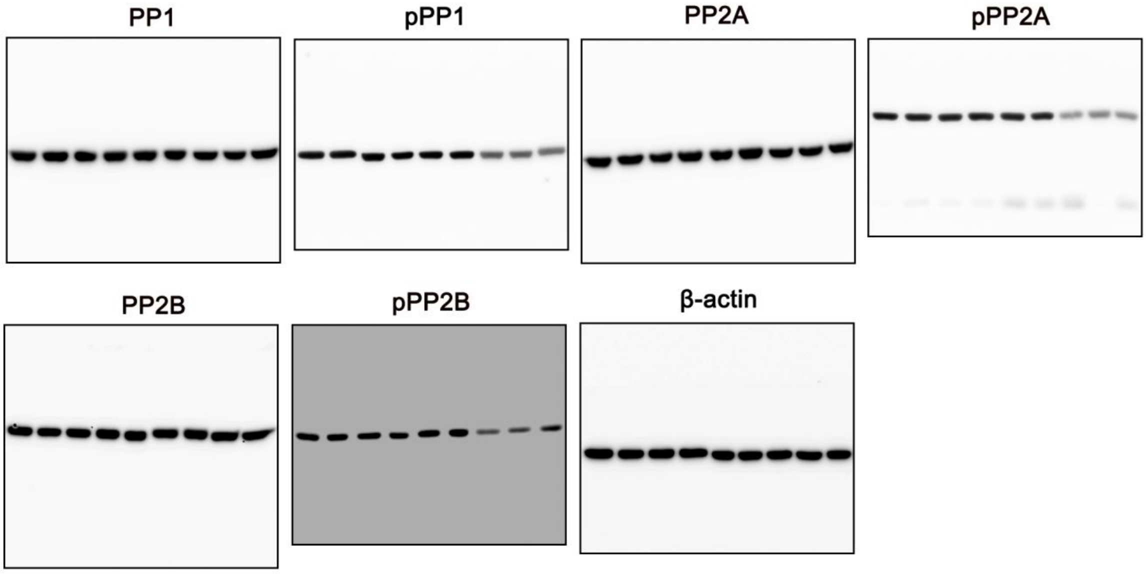

Supplementary Fig. 4. Full-length gel images of western blot data in Fig. 4.

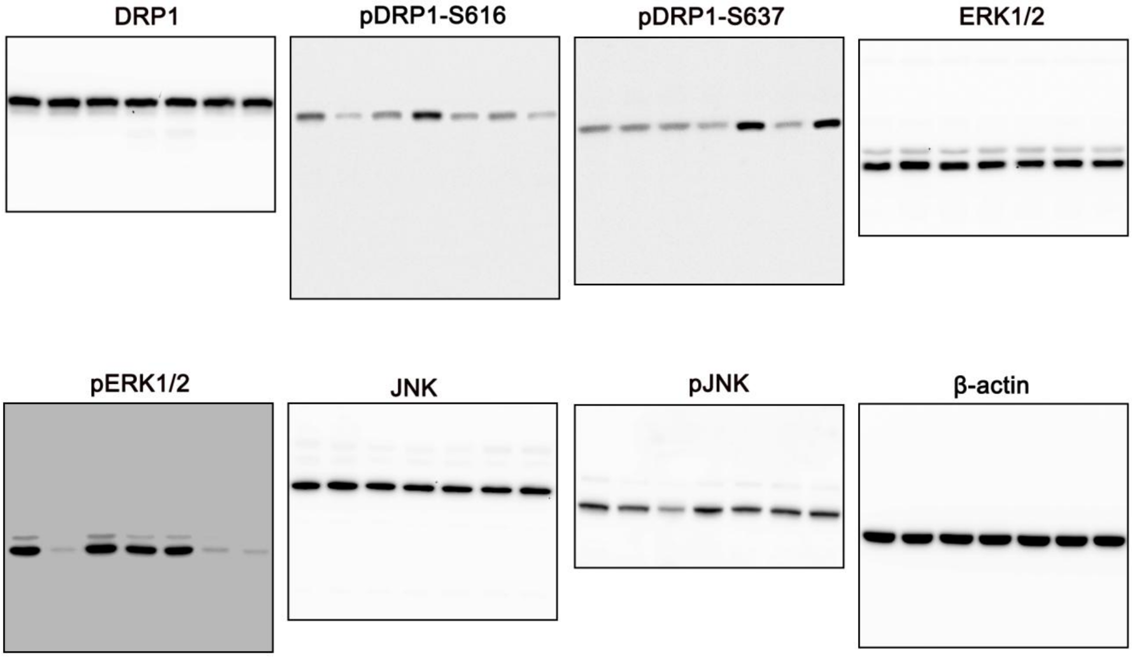

Supplementary Fig. 5. Full-length gel images of western blot data in Fig. 5.

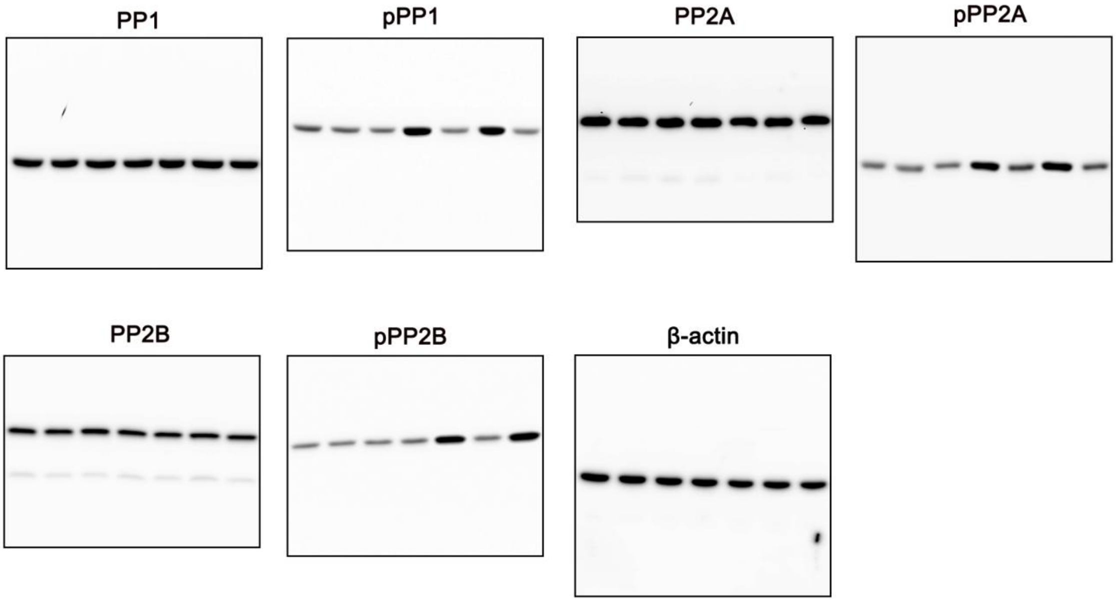

Supplementary Fig. 6. Full-length gel images of western blot data in Fig. 6.

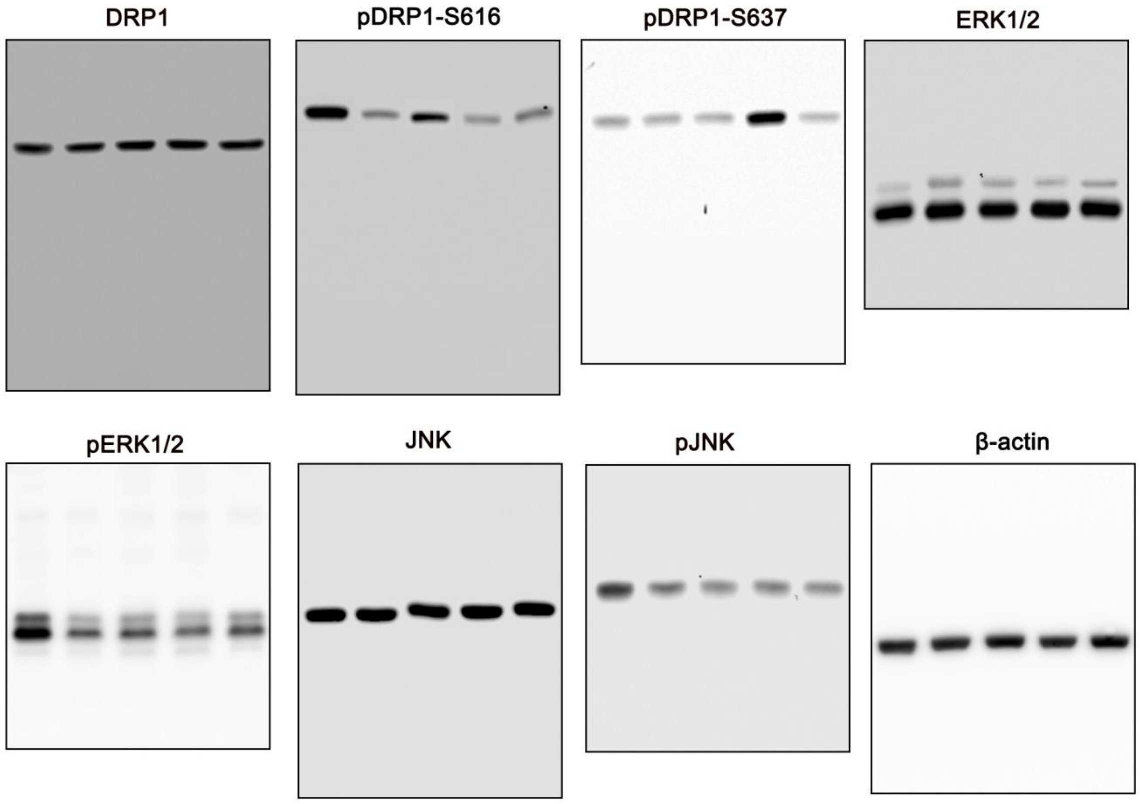

Supplementary Fig. 7. Full-length gel images of western blot data in Fig. 8.

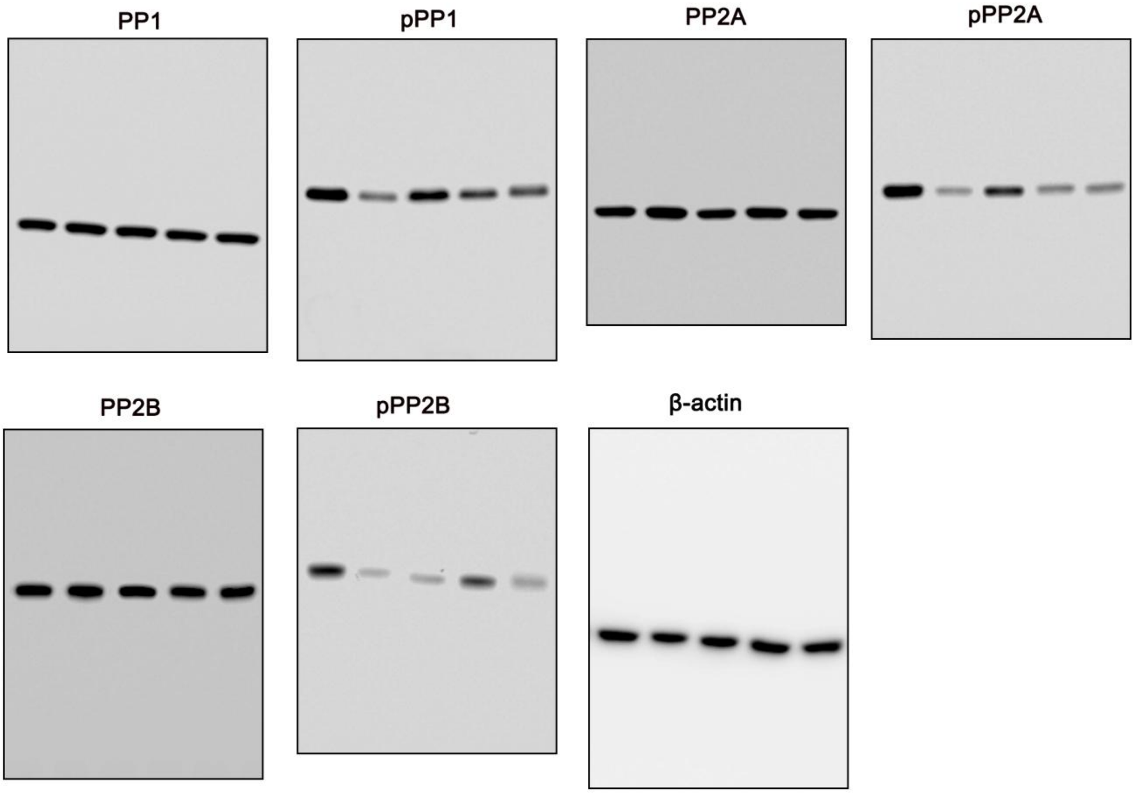

Supplementary Fig. 8. Full-length gel images of western blot data in Fig. 9.

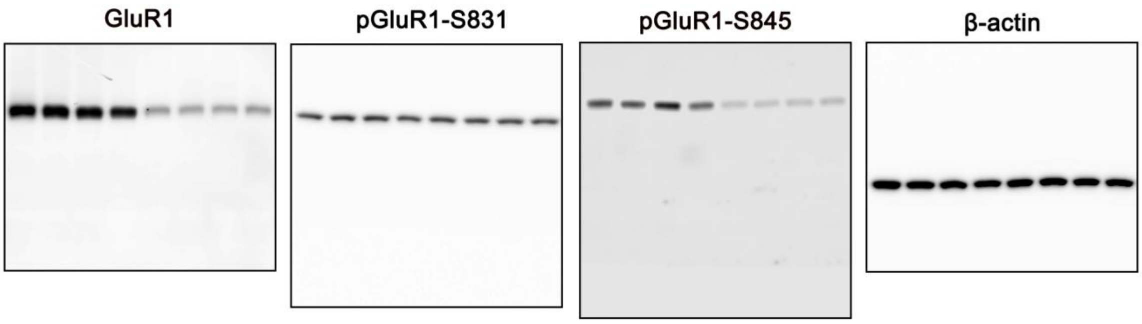

Supplementary Fig. 9. Full-length gel images of western blot data in Fig. 10.
